# Neurodegeneration in Parkinson’s disease: are we looking at the right spot?

**DOI:** 10.1186/s13041-025-01218-6

**Published:** 2025-08-01

**Authors:** Gabriel S. Rocha, Marco Aurelio M. Freire, Daniel Falcao, Tiago F. Outeiro, Rafael R. Lima, Jose Ronaldo Santos

**Affiliations:** 1 Department of Physiology, Federal University of Sergipe, Aracaju, 49107-230 Brazil; 2 Laboratory of Behavioral and Evolutionary Neurobiology, Department of Biosciences, Federal University of Sergipe, Itabaiana, SE 49500-000 Brazil; 3 Institute of Biological Sciences, Federal University of Pará, Belém, 66075-110 Brazil; 4 VCU Health Systems, Virginia Commonwealth University, Richmond, 23284 USA; 5 Department of Experimental Neurodegeneration, Center for Biostructural Imaging of Neurodegeneration, University Medical Center Göttingen, Göttingen, Germany; 6 Translational and Clinical Research Institute, Faculty of Medical Sciences, Newcastle University, Framlington Place, Newcastle Upon Tyne, NE2 4HH UK; 7 Max Planck Institute for Multidisciplinary Sciences, Göttingen, Germany; 8 Scientific Employee with an Honorary Contract at Deutsches Zentrum für Neurodegenerative Erkrankungen (DZNE), Göttingen, Germany

**Keywords:** Parkinsonism, Cell death, Axons, Dopamine, Neural transmission

## Abstract

Parkinson’s disease (PD) is recognized as the fastest-growing neurodegenerative disorder, impacting millions of individuals worldwide. It is primarily characterized by cardinal motor symptoms, including bradykinesia (slowness of movement), tremor, rigidity, and postural instability, which significantly impair the quality of life of those affected. Traditionally, the prevailing hypothesis has attributed these motor symptoms to the degeneration and subsequent loss of dopaminergic neurons in the *substantia nigra pars compacta* (SNpc). Currently, emerging research suggests that this neuron-centric view may be overly simplistic and not entirely accurate. In light of this, growing attention has turned to the role of axons within the nigrostriatal pathway—an extensive network connecting the *substantia nigra* to the striatum, essential for both dopamine transmission and the overall functioning of the motor control by the brain. By directing a focus toward this aspect, in this nano review article we examine why nigrostriatal axons deserve increased attention and should be considered a pivotal target for further therapeutic strategies in PD.

## Introduction

Parkinson’s disease (PD) is a progressive neurodegenerative disorder that affects around 10 million people worldwide [1]. Although PD includes both motor and non-motor symptoms, it is classically characterized by hallmark motor manifestations: bradykinesia, resting tremor, rigidity, and postural instability. These motor deficits are primarily attributed to the reduction of dopamine (DA) released from neurons projecting from the *substantia nigra pars compacta* (SNpc) to the striatum. The resulting dopaminergic deficiency in the dorsal striatum disrupts the balance of basal ganglia motor circuits, ultimately leading to bradykinesia [2]. From a cellular perspective, the primary histopathological feature of PD is the buildup of Lewy bodies, formed by aggregates of misfolded alpha-synuclein (α-syn). This accumulation is associated with neuroinflammation, oxidative stress, calcium (Ca^2+^) excitotoxicity, mitochondrial dysfunction, impaired lysosomal autophagy, and altered vesicle and protein trafficking [3].

It is traditionally postulated that the cardinal motor symptoms of PD begin when approximately 60–80% of the dopaminergic neurons in the SNpc have been lost [4]. Accordingly, experimental studies using distinct parkinsonism models have commonly focused on the density of tyrosine hydroxylase-positive (TH^+^) neuronal cell bodies in the SNpc as a parameter to assess the PD progression severity and correlate it with motor symptoms [5]. While this approach is not inaccurate, it overlooks additional aspects of neurodegeneration in PD. In light of this, recent studies have increasingly highlighted axonal degeneration as a key factor in PD progression. This line of research has gained prominence due to the potential impact of axonal degeneration on the emergence of motor symptoms associated with the disease [6, 7]. Understanding how axonal degeneration contributes to the broader pathological landscape of PD may yield valuable insights into mechanisms behind the onset and progression of motor impairments. By investigating specific changes that occur in axonal structure and their relationship to neuronal function, researchers aim to uncover new therapeutic targets that could mitigate or delay these debilitating symptoms.

## Why focus on axons?

For a long time, the concept of neurodegeneration centered essentially on neuronal soma death [8]. However, this view has since broadened to encompass synaptic dysfunction and disruption of neuronal connectivity [9]. In the context of PD, it is important to highlight key characteristics of SNpc axonal biology. First, neurons in this region possess long, unmyelinated axons, with extensive branching, allowing them to form numerous synapses with medium spiny neurons in the striatum. It is estimated that a single SNpc neuron can establish connections with around one million striatal neurons [2]. Due to this unique morphological feature, these neurons exhibit high bioenergetic demand [8]. The combination of elevated mitochondrial activity and dopamine metabolism at axonal terminals creates an environment prone to high oxidative stress, rendering these neurons particularly vulnerable to degeneration [2]. Another significant aspect to consider is the role of α-syn accumulation. Under physiological conditions, this protein is involved in synaptic vesicle recycling. Notwithstanding, in PD, it misfolds and accumulates within axonal terminals, disrupting synaptic function and leading to an increase in cellular oxidation through its interaction with DA, ultimately contributing to progressive neuronal degeneration [8].

Compelling evidence indicates that α-syn accumulation, a key pathological feature in PD, begins mainly in the axonal terminals, triggering a retrograde degenerative process toward the soma. Thus, when neuronal death begins to occur in the SNpc, nigral projections in the striatum may already be severely compromised [6]. Consistently, early co-accumulation of α-syn and synapsin III at synaptic terminals has been observed in the initial stages in a MPTP (1-methyl-4-phenyl-1,2,3,6-tetrahydropyridine)-induced mouse model of parkinsonism, thereby contributing to nigrostriatal denervation [10]. The same study also reported a notable reduction in the immunoreactivity of key proteins involved in dopaminergic signaling at early stages, specifically the DA transporter (DAT) and vesicular monoamine transporter 2 (VMAT2). These proteins play crucial roles in regulating DA levels within the striatum.

Strikingly, although VMAT2 is primarily recognized for its role in vesicular DA storage, it also contributes significantly to the packaging of gamma-aminobutyric acid (GABA) within the nigrostriatal pathway [11]. This suggests co-release of GABA and DA from SNpc projections to the striatum, as GABA release in the nigrostriatal terminals does not rely on the vesicular GABA transporter (VGAT), highlighting the importance of VMAT2 [12]. GABA release by SNpc dopaminergic neurons appears to play a crucial role for striatal neurons functioning and basal ganglia circuitry [12]. Moreover, it is involved in the autoregulation of phasic DA release through its binding to GABA_A_ receptors located on DA axons within the striatum. Notably, SNpc DA axons lack the molecular machinery required for GABA synthesis; instead, most GABA is taken up from neighboring striatal cells [13]. Taken together, these results underscore the pivotal role of processes carried out at DA SNpc axon terminals in modulating striatal and basal ganglia activity. Nevertheless, the mechanisms and implications of GABA/DA co-release in PD remain to be fully elucidated.

**Fig. 1:**
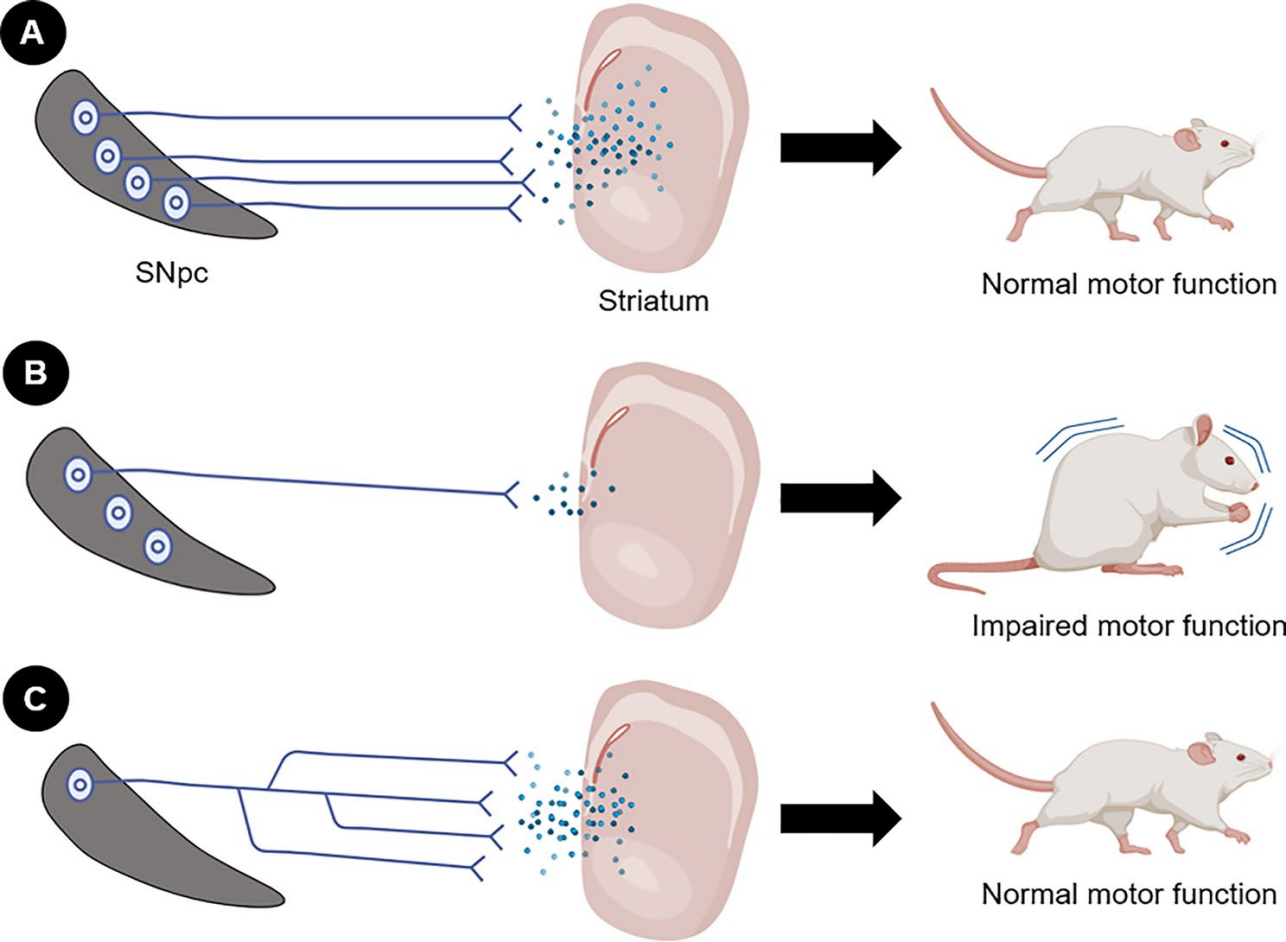
Role of nigrostriatal axon integrity for the maintenance of normal motor function. **(A)** Under physiological conditions, neurons and axons are preserved, sustaining neurotransmission and normal motor function. **(B)** Under pathological conditions such as PD, there is loss of cell bodies and, more notably, axonal degeneration, leading to reduced nigrostriatal neurotransmission and consequent motor impairment. **(C)** Surviving neurons in the *substantia nigra* undertake a compensatory mechanism to maintain striatal innervation. In this case, axonal sprouting occurs, preserving nigrostriatal neurotransmission and motor function

A recent study further supports for the role of axonal degeneration in the SNpc in the emergence of motor symptoms in PD [7]. The research focused on the impact of a mitochondrial DNA mutation–K320E-Twinkle^DaN^ – on dopaminergic neurons. This mutation significantly accelerates the processes involved in neuronal degeneration. Remarkably, after 20 months, mutant mice exhibited normal motor function, despite the loss of ∼70% of nigral dopaminergic neurons. The remaining neurons maintained ∼75% of axon terminals in the dorsal striatum, preserving normal neurotransmission. This maintenance of motor function was attributed to compensatory axonal sprouting from surviving neurons, which sustained striatal innervation (Fig. 1). Enhanced axon sprouting from the SNpc was associated with increased levels of unconventional neurotrophic factors, particularly netrin 1 (Ntn1) and ephrin-A2 (Efna2). In contrast, there were reduced levels of semaphorin 3 A (Sema3A) and Slit2, which are known to inhibit axon branching.

It is worth highlighting that in PD, α-syn pathology extends beyond the nigrostriatal pathway. Early accumulation of α-syn can occur in peripheral tissues and may spread in a “prion-like” manner from cell to cell via axons and synapses across neural circuits [13]. Recent studies have shown that pathological α-syn accumulates in the gut, liver [13], spinal cord, and kidneys [14] before overt neuronal damage occurs, suggesting early peripheral involvement. Alternatively, α-syn pathology may remain largely confined to the CNS, reflecting variability in both disease onset and progression.

## Conclusions and future perspectives

Emerging research suggests that axonal integrity within the nigrostriatal pathway may be even more critical than the survival of neuronal soma for maintaining motor function in neurodegenerative conditions such as PD. Given that soma and axon degeneration occur through distinct mechanisms [6], future therapeutic approaches for PD may prioritize protecting and promoting of axonal sprouting from the remaining SNpc neurons. By employing emerging gene therapy approaches, it will be possible to upregulate neurotrophic factor genes and downregulate genes that inhibit axon branching specifically in the nigrostriatal pathway, offering a more targeted and potentially effective treatment strategy than what is currently available.

Recent efforts to characterize PD through biological criteria represent a shift from symptom-based classification to approaches rooted in α-syn pathology, dopaminergic integrity, and genetic predispositions [4]—aligning with growing evidence of axonal involvement in early dysfunction. While this emerging view embraces prodromal mechanisms, a cautionary view stresses current limitations in diagnostic specificity and prognostic utility of biomarkers such as the synuclein seeding assay (SAA) [15].

## Data Availability

No datasets were generated or analysed during the current study.

## Abbreviations

PD: Parkinson’s disease
DA: Dopamine
SNpc: Substantia nigra pars compacta
Ca^2+^: Calcium
TH: Tyrosine hydroxylase
DAT: Dopamine
VMAT2: Vesicular monoamine transporter 2
VGAT: GABA transporter
GABA: Gamma-aminobutyric acid
α-syn: Alpha-synuclein

## References

[1] Bloem BR, Okun MS, Klein C. Parkinson’s disease. Lancet. 2021;397(10291):2284–303. 33848468 10.1016/S0140-6736(21)00218-X

[2] Giguère N, Burke Nanni S, Trudeau L-E. On cell loss and selective vulnerability of neuronal populations in Parkinson’s disease. Front Neurol. 2018;9:455. 29971039 10.3389/fneur.2018.00455 PMC6018545

[3] Morris HR, Spillantini MG, Sue CM, Williams-Gray CH. The pathogenesis of Parkinson’s disease. Lancet. 2024;403(10423):293–304. 38245249 10.1016/S0140-6736(23)01478-2

[4] Höglinger GU, Adler CH, Berg D, Klein C, Outeiro TF, Poewe W, Postuma R, Stoessl AJ, Lang AE. A biological classification of Parkinson’s disease: the synneurge research diagnostic criteria. Lancet Neurol. 2024;23:191–204. 38267191 10.1016/S1474-4422(23)00404-0

[5] Bové J, Perier C. Neurotoxin-based models of Parkinson’s disease. Neuroscience. 2012;211:51–76. 22108613 10.1016/j.neuroscience.2011.10.057

[6] Cheng HC, Ulane CM, Burke RE. Clinical progression in Parkinson disease and the neurobiology of axons. Ann Neurol. 2010;67(6):715–25. 20517933 10.1002/ana.21995 PMC2918373

[7] Paß T, Ricke KM, Hofmann P, Chowdhury RS, Nie Y, Chinnery P, et al. Preserved striatal innervation maintains motor function despite severe loss of nigral dopaminergic neurons. Brain. 2024;147(9):3189–203. 38574200 10.1093/brain/awae089

[8] Mishra AK, Dixit A. Dopaminergic axons: key recitalists in Parkinson’s disease. Neurochem Res. 2022;47:234–48. 34637100 10.1007/s11064-021-03464-1

[9] Wilson DM, Cookson MR, Bosch LVD, Zetterberg H, Holtzman DM, Dewachter I. Hallmarks of neurodegenerative diseases. Cell. 2023;186(4):693–714. 36803602 10.1016/j.cell.2022.12.032

[10] Serra M, Faustini G, Brembati V, Casu MA, Pizzi M, Morelli M, et al. Early α-synuclein/synapsin III co-accumulation, nigrostriatal dopaminergic synaptopathy and denervation in the MPTPp mouse model of Parkinson’s disease. Exp Neurol. 2025;383:115040. 39500391 10.1016/j.expneurol.2024.115040

[11] Tritsch NX, Ding JB, Sabatini BL. Dopaminergic neurons inhibit striatal output through non-canonical release of GABA. Nature. 2012;7419:262–6. 10.1038/nature11466 PMC3944587 23034651

[12] Patel JC, Sherpa AD, Melani R, Witkovsky P, Wiseman MR, O’Neill B, et al. GABA co-released from striatal dopamine axons dampens phasic dopamine release through autoregulatory GABAA receptors. Cell Rep. 2024;43(3):113834. 38431842 10.1016/j.celrep.2024.113834 PMC11089423

[13] Mather M. Autonomic dysfunction in neurodegenerative disease. Nat Rev Neurosci. 2025;26:276–92. 40140684 10.1038/s41583-025-00911-8

[14] Yuan X, Nie S, Yang Y, Liu C, Xia D, Meng L et al. 2025. Propagation of pathologic α-synuclein from kidney to brain may contribute to Parkinson’s disease. Nat Neurosci. 2025;28:577–588. 10.1038/s41593-024-01866-2 39849144

[15] Cardoso F, Schmidt P. 2025. Proposed biological definitions of Parkinson’s disease confuse understanding without delivering meaningful advances. J Parkinsons Dis. 2025;14:1877718X241308482. 10.1177/1877718X241308482 PMC13347491 39973501

